# The influence of strain on image reconstruction in Bragg coherent X-ray diffraction imaging and ptychography

**DOI:** 10.1107/S160057752100477X

**Published:** 2021-06-01

**Authors:** Chan Kim, Markus Scholz, Anders Madsen

**Affiliations:** a European X-ray Free-Electron Laser Facility, Holzkoppel 4, 22869 Schenefeld, Germany

**Keywords:** coherent X-ray diffraction imaging, Bragg ptychography, anti-phase domain boundary, strong phase object, modulus homogenization

## Abstract

A quantitative analysis of the effect of strain on phase retrieval in Bragg coherent X-ray diffraction imaging is reported. It is shown in reconstruction simulations that the phase maps of objects with strong step-like phase changes are more precisely retrieved than the corresponding modulus values. The simulations suggest that the reconstruction precision for both phase and modulus can be improved by employing a modulus homogenization (MH) constraint. This approach was tested on experimental data from a highly strained Fe–Al crystal which also features antiphase domain boundaries yielding characteristic π phase shifts of the (001) superlattice reflection. The impact of MH is significant and this study outlines a successful method towards imaging of strong phase objects using the next generation of coherent X-ray sources, including X-ray free-electron lasers.

## Abstract 2

A quantitative analysis of the effect of strain on image reconstruction in Bragg coherent X-ray diffraction imaging and ptychography reveals reconstruction artifacts caused by the limited spatial resolution. With the modulus homogenization constraint applied, the reconstruction artifacts are efficiently removed and an Fe–Al alloy sample with strong strain that features π phase steps due to anti-phase domain boundaries is successfully reconstructed.

## 1. Introduction   

Coherent X-ray diffraction imaging (CXDI) (Chapman & Nugent, 2010 ▸; Miao *et al.*, 2015 ▸), which can be regarded as an extension of X-ray crystallography (Sayre, 1980 ▸; Sayre *et al.*, 1998 ▸), has emerged as a new characterization tool in nano-science since the first successful experimental demonstration (Miao *et al.*, 1999 ▸). X-ray crystallography can provide 3D images of molecules with atomic resolution although the results are derived from averaged information (Pandey *et al.*, 2020 ▸; Gisriel *et al.*, 2019 ▸). Unlike crystallography, by CXDI it is possible to visualize both non-crystalline and crystalline objects and derive quantitative information relevant for their properties, such as the electron density, thickness, atomic composition, lattice displacement, atomic ordering, and defect information, depending on the experimental scheme (Miao *et al.*, 2003 ▸; Marathe *et al.*, 2010 ▸; Kim *et al.*, 2014 ▸, 2018*a* ▸; Robinson & Harder, 2009 ▸; Donnelly *et al.*, 2017 ▸; Mastropietro *et al.*, 2017 ▸).

The key point for a successful CXDI experiment is the phase retrieval process by which it is possible to reconstruct the lost phase information in reciprocal space and hence retrieve the image in real space. The first iterative phase retrieval algorithm was proposed by Gerchberg & Saxton (1972 ▸) and later refined to yield the error reduction (ER) and hybrid input output (HIO) algorithms by Fienup (1978 ▸, 1982 ▸, 1993 ▸). These developments and the first successful CXDI demonstration on experimental data generated tremendous interest and stimulated a wealth of activity leading to novel algorithms and approaches, *e.g.* shrinkwrap, guided HIO (GHIO), and the ptychographical iterative engine (PIE) (Marchesini *et al.*, 2003 ▸; Chen *et al.*, 2007 ▸; Thibault *et al.*, 2009 ▸). Despite the impressive achievements there is still need for improvement, especially concerning the best resolution that can be achieved as well as reliable phase retrieval from samples with strong phase variations. The finite pixel size of detectors increases the reconstruction error and distorts the retrieved image which presents a particular problem for Bragg CXDI from crystalline samples with large strain (Ihli *et al.*, 2016 ▸; Cha *et al.*, 2010 ▸). Also, there are several reports about successful reconstructions of strong phase objects using the ptychography method, but sharp edge structures are often blurred and unphysical phase and modulus fluctuations are observed (Esashi *et al.*, 2018 ▸; Mochi *et al.*, 2020 ▸).

In this paper we study the reconstructed image quality, both modulus and phase, as a function of the strength of phase variations with the aim of improving the accuracy of imaging for highly strained crystalline samples. The reconstruction error in reciprocal space becomes larger as the phase variation over the sample increases and this is related to symmetry breaking of the diffraction intensities. The phase appears to be more accurately retrieved than the modulus but both quantities can be refined by employing a modulus homogenization constraint (Godard *et al.*, 2011 ▸; Kim *et al.*, 2018*b* ▸).

## 2. Numerical simulation   

**Figure 1:**
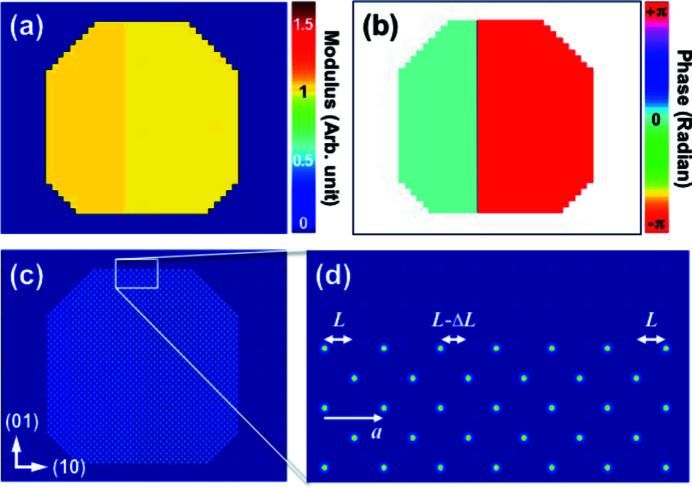
Two different numerical models employed for quantitative analyses of the effect of strain. (*a*, *b*) General phase model (GPM) which assigns both a modulus (*a*) and a phase (*b*) value to each pixel. (*c*, *d*) Real atomistic model (RAM) which contains only modulus values. Here the phase shift in Bragg diffraction originates from lattice distortions. *L* and Δ*L* in (*d*) correspond to half of the lattice constant (*a*/2) in the (10) crystal plane direction and the step-like displacement, respectively. *L* was fixed to 32 pixels and Δ*L* was varied for the different simulations (see Table 1 ▸).

Two different models, denoted the general phase model (GPM) and the real atomistic model (RAM), are used for a quantitative analysis (see Fig. 1 ▸). The former has been widely used and assigns both a modulus and a phase value to each pixel, while the latter is a more realistic crystal structure model with modulus values corresponding to the electron density. Hence, the RAM is particularly suited for diffraction imaging simulations in Bragg geometry. The GPM features a sharp (one pixel wide) phase boundary with a phase step that was varied from 0 to 2π rad in the simulations. Examples of modulus and phase images with a π phase step are shown in Figs. 1 ▸(*a*) and 1(*b*). The RAM has a smoother boundary because its phase step originates from a gradual variation of the lattice constant *a* in the transition zone. The total amount of atomic displacement (see Table 1 ▸) yields the phase step between two regions and can also be varied in the simulations to attain values from 0 to 2π rad [see Figs. 1 ▸(*c*) and 1(*d*) for an example with a resulting π phase step].

**Figure 2:**
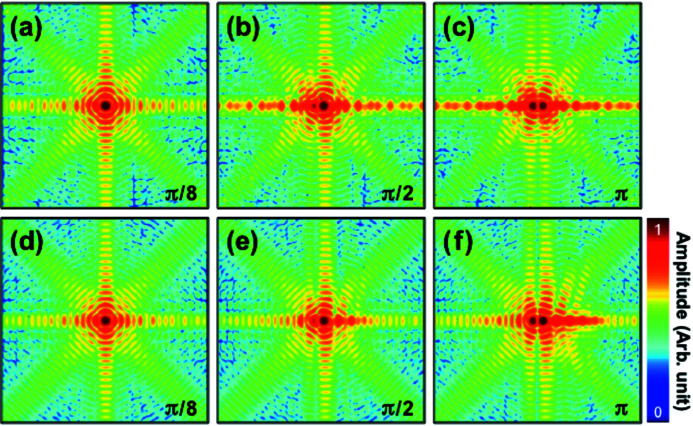
(*a*, *b*, *c*) Calculated diffraction amplitudes of the GPM with three different phase steps, π/8 (*a*), π/2 (*b*), and π (*c*). (*d*, *e*, *f*) Calculated diffraction amplitudes of the RAM with phase steps of π/8 (*d*), π/2 (*e*), and π (*f*).

Diffraction amplitudes of both models with varying phase steps were calculated and three representative diffraction amplitudes with π/8, π/2, and π phase steps are displayed in Fig. 2 ▸. Due to the crystal-like periodic structure of the RAM, many different Bragg peaks are obtained by a fast Fourier transformation (FFT) of the structure. Here, we focus on the (20) Bragg peak of the RAM because it corresponds to the lattice displacement direction indicated in Figs. 1 ▸(*c*) and 1(*d*). The array size of the diffraction patterns is 320 × 320 pixels with an oversampling ratio of about 10 in each dimension. As the phase step increases, the diffraction patterns become less symmetric. The GPM with π phase step [Fig. 2 ▸(*c*)], however, has again a symmetric diffraction pattern due to the strong phase gradient (one pixel discontinuity). This is not the typical behavior of a strongly strained lattice and indicates that the GPM is less suitable for simulating Bragg CXDI which is our focus here.

## 3. Result and discussion   

### 3.1. Effect of limited spatial resolution in the phase retrieval process   

To study how limited spatial resolution leads to averaging and distorts the retrieved real space images of strained objects, only the central 160 × 160 pixels was used for image reconstruction, *i.e.* half the size in each dimension. As a result, we expect averaging over four neighboring pixels in the retrieved real space image together with an ambiguity in the location of the phase step, typically half a pixel of the reconstructed image. The HIO algorithm, one of the most widely used algorithms in Bragg CXDI, was employed for all image reconstructions. To avoid effects other than those originating from reduced array size, typical experimental features like missing center pixels or detector noise were not incorporated.

**Figure 3:**
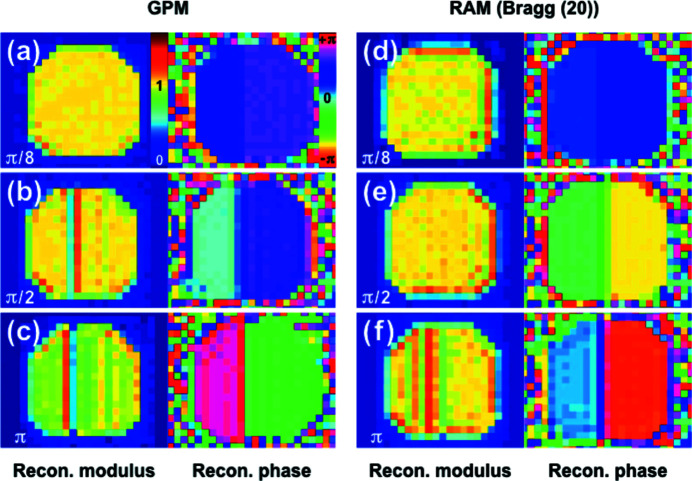
(*a*, *b*, *c*) Reconstructed modulus (left column) and phase (right column) of GPM with π/8 (*a*), π/2 (*b*), and π (*c*) phase steps. (*d*, *e*, *f*) Reconstructed modulus (left column) and phase (right column) of RAM with π/8 (*d*), π/2 (*e*), and π (*f*) phase steps. Only the central 160 × 160 array is used for image reconstruction. One representative image out of 30 independent reconstructions is shown for each simulation.

Phase retrieval of the diffraction intensities is performed from both models with varying phase steps, ranging from 0 to 2π rad. Three representative real space images of both the GPM and the RAM are shown in Fig. 3 ▸. Left and right sides in panels (*a*)–(*f*) correspond to the reconstructed moduli and phases, respectively. The averaging effect, resulting in a modulus cavity near the phase step, is clearly visible in the modulus images, especially in Figs. 3 ▸(*b*), 3(*c*) and 3(*f*), as the phase step increases and results in a small artificial fluctuation near the phase step. By averaging two complex values with same modulus and a π phase offset, *e.g.* *A*exp(*ix*) and *A*exp[*i*(*x* + π)], the averaged modulus value becomes zero and will strongly affect the reconstruction because any phase value can be a solution. Hence, we expect a problem for precise reconstruction around a sharp π phase step due to averaging. This is also illustrated by the fact that the RAM modulus images are more accurately retrieved than those of the GPM, compare for instance Figs. 3 ▸ (*b*) and 3(*e*), because the RAM has a smoother phase boundary.

To perform a quantitative analysis of the effect of finite pixel size and averaging we evaluated the symmetry index (SI) values of the calculated diffraction amplitudes, the reconstruction errors (*R*_err_) in reciprocal space, and the similarity (*R* values) of reconstructed images in real space. The SI is defined as 

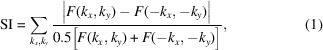

where *F*(*k*_*x*_, *k*_*y*_) is the calculated diffraction amplitude and *k*_*x*_, *k*_*y*_ are the coordinates in reciprocal space. The cases SI = 0 and SI ≥ 1 indicate full symmetry and asymmetry, respectively, while the case 1 > SI > 0 indicates partial symmetry (Błażkiewicz *et al.*, 2014 ▸). *R*_err_ is comparing the reconstructed amplitude with the initial input amplitude and is given as 

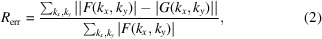

where *G*(*k*_*x*_,*k*_*y*_) is the final reconstructed amplitude. The convergence of the iterative algorithm is also evaluated on the reconstructed real space images by using *R* values (Song *et al.*, 2008 ▸) as 

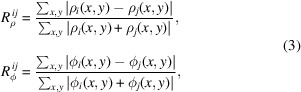

where ρ and ϕ are the reconstructed modulus and phase, respectively, and indices *i* and *j* indicate the final result of the *i*th and *j*th independent reconstructions with random initial phases.

**Figure 4:**
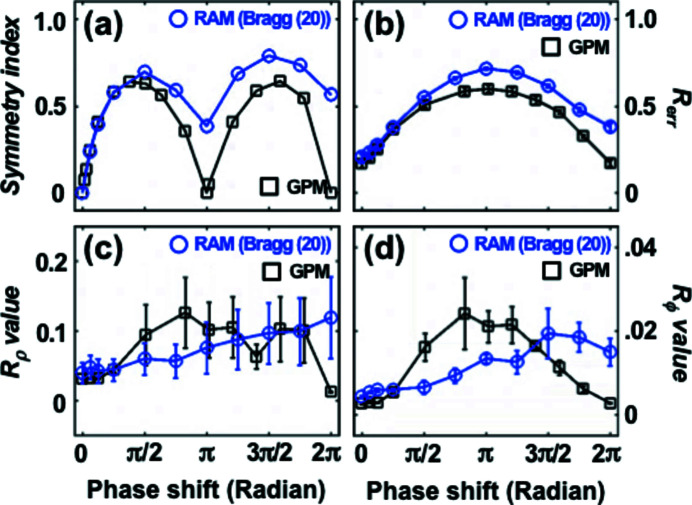
(*a*) SI value of the calculated diffraction amplitudes (Fig. 2 ▸) as a function of phase shift. (*b*) Averaged *R*_err_ after 30 independent reconstructions as a function of phase shift. (*c*, *d*) *R*_ρ_ value (*c*) and *R*_ϕ_ value (*d*) which were calculated as an average of the five best images out of 30 independent reconstructions.

The SI calculation was applied to the simulated diffraction amplitudes and the reconstructed reciprocal and real space images were evaluated using *R*_err_ and *R* values (Fig. 4 ▸). Fig. 4 ▸(*a*) shows the SI calculation and the diffraction amplitude is fully symmetric at a phase step of 0 (no phase step). As the value increases, the SI also increases steadily although small dips are observed near π and 2π. This happens because the central speckle of the diffraction amplitude is split into two [Figs. 2 ▸(*c*) and 2(*f*)] near a π phase step and the overall amplitudes become more symmetric. However, the reconstruction is more difficult in this case which is illustrated by the *R*_err_ value shown in Fig. 4 ▸(*b*). Figures 4 ▸(*c*) and 4(*d*) show *R* values averaged over the five best images out of 30 independent reconstructions. The five best images were selected based on complete *R* values calculated with 30 independent reconstructions. Ten *R* values calculated from the five best images were used for Figs. 4 ▸(*c*) and 4(*d*). A plateau region between 0 and π/8 can be found for both *R* values, which can be considered as the weak phase object region. Here, we expect almost no artificial phase modulations near the phase step on the reconstructed images. Upon increasing the phase step, the *R* values keep increasing although the *R*_ϕ_ value of the GPM decreases above π when a 2π phase step is approached (phase wrapping). The *R*_ρ_ values have a maximum of ∼0.1 (∼10% difference) while the maximum *R*_ϕ_ value is only around 0.02 (2% difference) in the investigated phase shift range. This result indicates that the reconstructed phase values are quite reliable compared with the modulus values but still the reconstruction error in reciprocal space (*R*_err_) can be quite large.

### 3.2. Modulus homogenization constraint   

Unfortunately, working with limited spatial resolution is unavoidable in atomic systems because the current best image resolution obtained in CXDI is in the nanometre range (Shapiro *et al.*, 2014 ▸; Takahashi *et al.*, 2013 ▸) which is much bigger than the atomic length scale relevant for lattice strain and hence for Bragg CXDI. This effect, however, can be minimized by guiding the reconstructed modulus to attain realistic values, also known as the modulus homogenization (MH) constraint (Godard *et al.*, 2011 ▸; Kim *et al.*, 2018*b* ▸). The MH constraint smoothens the reconstructed modulus image by which a more accurate result is achieved, for instance avoiding zero moduli values of the object caused by averaging over a π phase step as discussed in Section 3.1. Therefore, the MH constraint leads to more accurately retrieved phase values depending on the object structure.

In the simulations, we used a Gaussian smoothing function of 1σ as MH constraint. After a certain amount of phase retrieval iterations, Gaussian smoothing was applied to the reconstructed modulus values for each iteration. The phase retrieval process together with the MH constraint is then continued until the reconstruction is stabilized. In the current study, MH was applied after 200 HIO iterations with a total of 10000 iterations performed, although the error and images were approximately stabilized after about a thousand iterations. The *R*_err_ value slightly increases with the MH applied since it is pushing the reconstruction away from a simple least-square optimization. Similar effects have been observed with other real-space constraints, *e.g.* positivity (Elser & Millane, 2008 ▸). Convergence of the algorithm measured by stagnation of the *R*_err_ value, however, is still a valid criterion.

**Figure 5:**
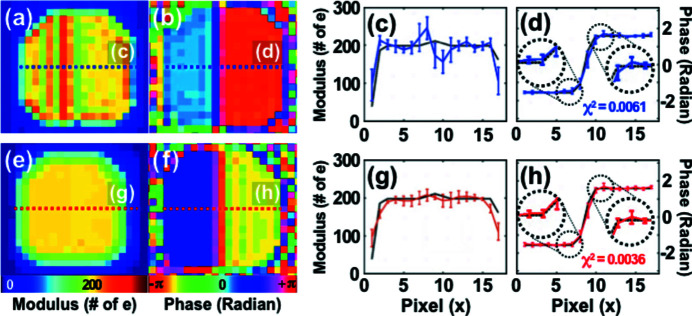
(*a*, *b*) Reconstructed modulus (*a*) and phase (*b*) of the RAM object with a π phase step without the MH constraint applied. (*c*, *d*) Line profile of the reconstructed modulus (*c*) and phase (*d*), shown by blue dotted lines in (*a*) and (*b*), respectively. The five best reconstructions were averaged for the line profile analysis and the black solid lines show the original modulus and phase. (*e*, *f*) Reconstructed modulus (*e*) and phase (*f*) of the RAM object with π phase step using the MH constraint. (*g*, *h*) Line profile of the reconstructed modulus (*g*) and phase (*h*), shown by red dotted lines in (*e*) and (*f*), respectively. The five best reconstructions are averaged for the line profile analysis where the black solid lines show the original modulus and phase.

Phase retrieval with the Gaussian MH constraint was applied to reconstruct the π phase-stepped RAM object [Fig. 2 ▸(*f*)]. Fig. 5 ▸ shows the results of two independent image reconstructions without [Figs. 5 ▸(*a*)–5(*d*)] and with [Figs. 5 ▸(*e*)–5(*h*)] the MH constraint. As shown in Fig. 5 ▸(*e*), the unwanted averaging effect near the phase step is clearly improved compared with Fig. 5 ▸(*a*). A good modulus image is not achieved for all reconstructions (all random starts), but 14 good images (without modulus cavity) were achieved out of 100 independent reconstructions. The reconstructed phase image shown in Fig. 5 ▸(*f*) is also improved compared with Fig. 5 ▸(*b*). For a quantitative analysis of the phase retrieval accuracy, line profiles of both images are plotted in Figs. 5 ▸(*d*) and 5(*h*) and compared with the original phase (black solid lines). The five best phase images based on an *R* value evaluation of 14 good images are averaged after a baseline correction. Chi-square (χ^2^) values, by comparing the reconstructed phases with the phase of the original model, are calculated in both cases as shown in Figs. 5 ▸(*d*) and 5(*h*) and an improvement of almost a factor of two (from 0.0061 to 0.0036) is observed. The improvement of the reconstructed phase is significant since a 10^−4^ strain sensitivity is often discussed in Bragg coherent diffraction imaging (Carnis *et al.*, 2019 ▸; Leake *et al.*, 2019 ▸).

### 3.3. Application of MH constraint to Bragg ptychography   

To test whether the MH constraint is also useful and applicable to real experimental data, a B2-ordered Fe–Al alloy crystal was investigated by 3D Bragg ptychography. Ptychography is an extension of CXDI introducing an additional overlap constraint in real space but is otherwise similar so the MH constraint can be used as discussed. The sample is highly strained but also contains antiphase domain boundaries (ADBs) with associated π phase steps (Kim *et al.*, 2018*a* ▸). Near the ADBs, additional phase variations are expected due to lattice distortions and atomic rearrangements. The experiment was performed at beamline ID01 of ESRF – The European Synchrotron, France (Chahine *et al.*, 2014 ▸). The X-ray energy was 7 keV selected by a Si(111) monochromator and the beam focused to about 180 nm (H) × 70 nm (V) by a Fresnel zone plate. A MAXIPIX detector with 512 (H) × 512 (V) pixels of 55 µm pixel size was used to record data sets of the Fe–Al alloy crystal. The detector was mounted on the 2θ arm of the diffractometer with a sample-to-detector distance of 1.2 m.

**Figure 6:**
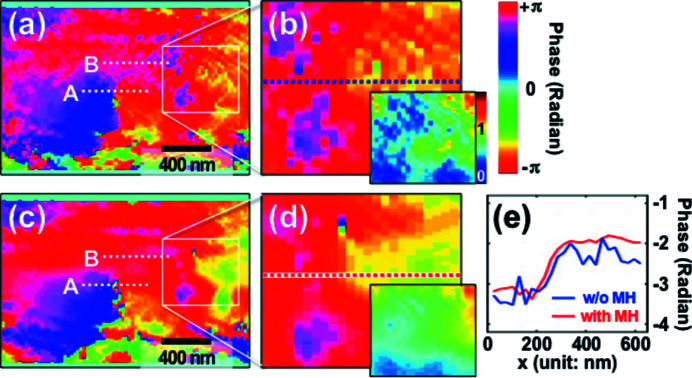
(*a*, *b*) Central pixel slice (sectioned) of the reconstructed (001) phase image (voxel) of an Fe–Al alloy crystal (B2 phase) without the MH constraint at different magnifications. (*c*, *d*) Same as above but with the MH constraint applied. The insets in (*b*) and (*d*) show reconstructed modulus images. (*e*) Line profiles of the reconstructed phases marked by a blue dotted line in (*b*) and a red dotted line in (*d*). The phase profiles along the lines denoted A and B in (*a*) and (*c*) are plotted in Fig. 7 ▸.

**Figure 7:**
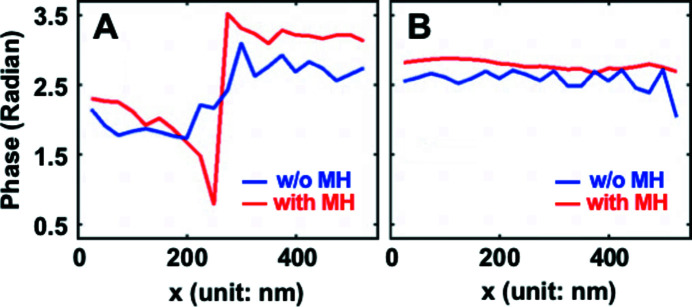
Phase line profiles from Figs. 6 ▸(*a*) and 6(*c*). A is the line profile across an ADB while B corresponds to a monodomain, see white dotted lines in Figs. 6 ▸(*a*) and 6(*c*). In the former case the step is sharpened by the MH constraint while in the latter case a smoothening is the result.

A full 3D Bragg ptychography data set was taken on the (001) superlattice reflection with θ_001_ = 17.55°, which is sensitive to both the ADBs and general lattice strain (Marcinkowski & Brown, 1962 ▸; Stadler *et al.*, 2007 ▸). Further details about the Bragg ptychography experiment are given by Kim *et al.* (2018*a* ▸). A combination of two different methods, namely the ordered subset (OS) and conjugated gradient (CG) algorithms, was applied as the main CXDI phase retrieval algorithm in the ptychographic reconstruction (Godard *et al.*, 2012 ▸) and the effect of the MH constraint was evaluated. Here, we used a 3D Gaussian smoothing function of 3σ and the MH constraint was applied in the second half of a sequence of 4000 iterations. We selected 3σ for the Bragg ptychography data analysis instead of 1σ used for the numerical analysis since the Fe–Al alloy sample structure is much more complex. Larger σ values result in broadening of the reconstructed modulus, especially near boundaries, but the reconstructed phase is still significantly improved by the MH constraint. Figure 6 ▸ shows results of reconstructed phase images of the Fe–Al alloy sample without and with the MH constraint. Without the MH constraint [Figs. 6 ▸(*a*) and 6(*b*)] clear phase oscillations are observed similar to the numerical examples discussed earlier. In addition, the modulus is very discontinuous and attains zero values which is not expected for this sample. With the MH constraint applied [Figs. 6 ▸(*c*) and 6(*d*)] the phase fluctuations are clearly reduced and much smoother phase variations are obtained inside domains which would be expected for crystalline samples. Also, the modulus image is greatly improved. Figure 7 ▸ highlights the difference between the phase variation inside a domain and over an ADB. Again, the impact by applying the MH constraint is significant in both cases guiding the reconstruction towards the expected behavior.

## 4. Conclusion   

In summary, we have addressed the problem of averaging by limited spatial resolution and the effect it has on a correct image reconstruction in Bragg diffraction geometry, particularly concerning imaging of strong phase objects for instance caused by strain of the atomic lattice or antiphase domains. It is investigated whether the MH constraint is applicable to the problem and we conclude that it provides much better image quality both in simple CXDI simulations of phase steps and for experimental ptychography data. In the simulations a limited spatial resolution mostly results in errors of the reconstructed modulus but also yields small phase fluctuation artifacts near abrupt phase steps. The MH constraint successfully deals with this problem. In the simulations, the reconstructed phase images in real space are reliable (*R*_ϕ_ value < 2% error for all the investigated phase steps) but the reconstructed modulus becomes rather inaccurate (*R*_ρ_ value ≃ 10% difference) as the phase step increases. With the MH constraint applied, the limited spatial resolution effect on the modulus can be minimized and the reconstructed phase is improved by almost a factor of two (χ^2^ evaluation). An Fe–Al crystalline sample featuring lattice strain and π phase steps due to ADBs was successfully reconstructed with the MH constraint and here it shows extraordinary impact on the results. The MH constraint is very efficient in correcting the reconstructions and removing artifacts both in phase and modulus that otherwise could be mistaken for sample structure. This work paves the road towards a better understanding of phase retrieval and image reconstruction in Bragg geometry of crystalline samples with large phase shifts originating from step-like atomic changes. This paper discusses the effect of the MH in detail, which will be useful for the community in deciding whether to use MH or not.

## Figures and Tables

**Table 1:** Phase steps of the RAM with respect to the (20) Bragg peak

Displacements (Δ*L*) in pixels	Total displacement in pixels	Total phase step in radians
1	1	π/16
1, 1	2	π/8
1, 1, 1, 1	4	π/4
1, 1, 1, 1, 1, 1, 1, 1	8	π/2
1, 1, 1, 2, 2, 2, 2, 1	12	3π/4
1, 2, 2, 2, 2, 2, 2, 2, 1	16	π
1, 1, 2, 4, 4, 4, 2, 1, 1	20	5π/4
2, 2, 4, 4, 4, 4, 2, 2	24	3π/2
2, 4, 4, 4, 4, 4, 4, 2	28	7π/4
2, 4, 4, 4, 4, 4, 4, 4, 2	32	2π
